# More than Mucositis: Pediatric RIME Following Co-Infection with SARS-CoV-2 and *Mycoplasma pneumoniae*—A Case Report and Mini-Review

**DOI:** 10.3390/idr17050121

**Published:** 2025-09-24

**Authors:** Alina Corina Grama, Ovidiu Grama, Măriuca Mănescu, Mihaela Chinceșan

**Affiliations:** 1 Department of Pediatrics, Faculty of Medicine, George Emil Palade University of Medicine, Pharmacy, Science, and Technology of Târgu Mureș, 38 Gheorghe Marinescu, 540142 Târgu Mureș, Romania; alina.grama@umfst.ro (A.C.G.); mihaela.chincesan@umfst.ro (M.C.); 2 Department of Pediatrics, Emergency County Clinical Hospital of Târgu Mureș, 50 Gheorghe Marinescu, 540136 Târgu Mureș, Romania; 3 Department of Obstetrics and Gynecology 2, Faculty of Medicine, George Emil Palade University of Medicine, Pharmacy, Science, and Technology of Târgu Mureș, 38 Gheorghe Marinescu, 540142 Târgu Mureș, Romania; ovidiu.grama@umfst.ro; 4 Doctoral School, George Emil Palade University of Medicine, Pharmacy, Science, and Technology of Târgu Mureș, 38 Gheorghe Marinescu, 540142 Târgu Mureș, Romania

**Keywords:** reactive infectious mucocutaneous eruption (RIME), *Mycoplasma pneumoniae*, SARS-CoV-2, pediatric mucositis, coinfection

## Abstract

**Background:** Reactive Infectious Mucocutaneous Eruption (RIME) is a mucositis-predominant syndrome that usually follows respiratory infections in children. Although *Mycoplasma pneumoniae* is a well-established trigger, viral pathogens as triggers—especially SARS-CoV-2—have been increasingly reported. RIME is often misclassified as Stevens–Johnson syndrome (SJS), which may lead to inappropriate management. **Case Presentation:** We describe a 12-year-old previously healthy boy who presented with fever, dry cough, odynophagia, and vomiting for 9 days. On admission, he had severe oral ulcerations, bilateral conjunctivitis, and a non-blanching maculopapular rash. Laboratory tests confirmed co-infection with *M. pneumoniae* and SARS-CoV-2. Inflammatory markers were mildly elevated. Notably, the patient also developed asymptomatic sinus bradycardia, with no signs of structural heart disease. He was treated with antibiotics, intravenous corticosteroids, and supportive care. His mucosal symptoms improved rapidly, and he was discharged in stable condition on day 7. Follow-up at 12 days showed near-complete resolution of all lesions. **Conclusions:** This case illustrates several clinically relevant features. First, it highlights a dual infectious trigger—*M. pneumoniae* and SARS-CoV-2—that may have contributed to a more severe mucosal reaction. Second, the patient developed transient sinus bradycardia without myocardial involvement, suggesting a possible inflammatory autonomic response, rarely reported in RIME. Finally, this case supports the early use of corticosteroids in severe mucosal disease, with good outcomes and no complications. Prompt recognition of RIME, especially in the context of viral–bacterial coinfection, is essential to avoid misdiagnosis and to guide appropriate, multidisciplinary management.

## 1. Introduction

*Mycoplasma* species are among the smallest known free-living organisms, typically measuring 0.2 to 0.3 μm in diameter. Despite their minimal size, these bacteria are of significant clinical relevance, with over 100 identified species capable of causing a broad spectrum of infections and associated symptoms [1]. Among them, *Mycoplasma pneumoniae* is particularly noteworthy as a major etiological agent of atypical pneumonia, commonly referred to as “walking pneumonia.” This condition affects individuals across all age groups and is prevalent in both pediatric and adult populations [2]. However, it often remains underdiagnosed, primarily due to the limited use of microbiological testing in routine clinical practice. As a result, many infections go undetected, delaying appropriate treatment [3].

While *M. pneumoniae* is primarily associated with respiratory tract infections, it is also implicated in a range of extrapulmonary manifestations. These include serious complications such as meningoencephalitis, myocarditis, pericarditis, hemolytic anemia, nephritis, and various dermatological conditions [4,5]. Mucocutaneous involvement, which may arise from systemic inflammatory responses, is observed in up to 25–33% of affected individuals and can lead to lasting sequelae [6]. Serious skin and mucosal conditions related to *M. pneumoniae* include Stevens–Johnson syndrome (SJS), erythema multiforme, and Reactive Infectious Mucocutaneous Eruption (RIME) [7].

RIME is a severe mucocutaneous reaction that occurs post-infection, primarily affecting children after respiratory illnesses. This condition is characterized by significant mucositis, impacting the oral, ocular, and anogenital regions, while skin involvement is typically limited. Formerly referred to as *Mycoplasma pneumoniae*-Induced Rash and Mucositis (MIRM), this syndrome is now recognized to have a broader etiological spectrum. Recent evidence has demonstrated its association not only with *Mycoplasma pneumoniae* but also with other infectious agents, including *Chlamydia pneumoniae*, group A *Streptococcus*, and various viral pathogens such as adenovirus, influenza, and parainfluenza viruses [8,9].

This report presents the case of a 12-year-old boy who developed RIME following infections with both *Mycoplasma pneumoniae* and SARS-CoV-2. Description of this case underscores the challenges of diagnosing and treating RIME, particularly when both viral and bacterial infections are present.

## 2. Case Presentation

A previously healthy 12-year-old boy was admitted to a regional hospital with a 9-day history of high-grade fever, persistent dry cough, repeated vomiting, and progressively worsening odynophagia. Despite antipyretics and supportive care at home, symptoms persisted, and on presentation the patient appeared ill, fatigued, and mildly dehydrated. Clinical examination revealed hemorrhagic crusting of the lips, a markedly erythematous pharynx, conjunctival injection, and a fleeting erythematous rash. Respiratory distress requiring low-flow oxygen supplementation and sinus bradycardia (44 beats per minute) were also noted.

Laboratory evaluation demonstrated a moderate inflammatory response, with a C-reactive protein (CRP) of 23.82 mg/L. Given the fever, cough, and systemic findings, the case was initially interpreted as community-acquired pneumonia, and intravenous cefotaxime was initiated as part of empiric management, in accordance with pediatric CAP guidelines supporting third-generation cephalosporin use in severe or unclear cases. As symptoms worsened and RT-PCR returned positive for SARS-CoV-2, the therapeutic regimen was expanded beyond rehydration to include intravenous hydrocortisone hemisuccinate to address systemic inflammation, fluconazole for antifungal prophylaxis, gastric protection to reduce the risk of steroid-induced gastrointestinal complications, and inhaled bronchodilators to relieve respiratory distress. Antiviral therapy was attempted with Nirmatrelvir/ritonavir, but administration was discontinued due to intolerance (nausea and vomiting), and remdesivir was unavailable. Despite these measures, the patient’s respiratory and mucocutaneous symptoms progressed, prompting transfer to our tertiary facility.

On arrival, he was afebrile (35.7 °C) but appeared pale, ill, and further weakened from decreased oral intake. There was no history of medication exposure prior to illness onset, no allergies, and no autoimmune disease. Past medical and family history were unremarkable. Immunizations were up to date according to the national mandatory vaccination schedule, except for the COVID-19 vaccination.

**Figure 1:**
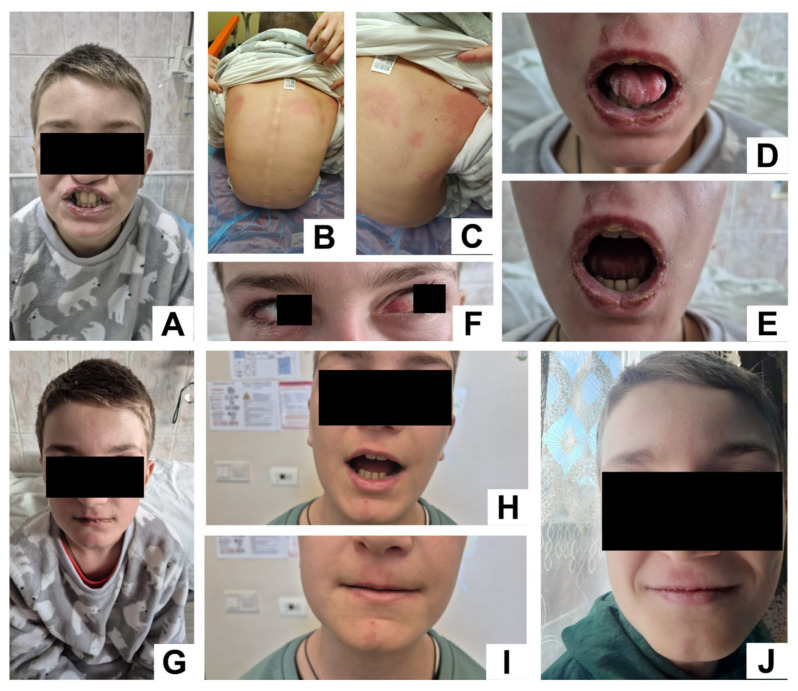
Panels (**A**–**C**) depict the early clinical presentation during the first days of admission, showing oral mucositis and scattered erythematous skin lesions. Panels (**D**–**F**), taken on day 3 of hospitalization, highlight bilateral conjunctivitis and worsening oral lesions. Panel (**G**) (discharge day) shows marked improvement in mucosal and cutaneous findings. Panels (**H**–**J**), obtained 12 days post-discharge, demonstrate near-complete resolution of lesions, confirming favorable recovery.

Physical examination revealed severe oral mucositis with painful ulcerations, hemorrhagic crusting of the lips, and erythematous oropharynx. Bilateral conjunctival injection with mucopurulent discharge consistent with bacterial conjunctivitis was noted. Skin examination revealed multiple scattered erythematous macules and thin plaques over the trunk and flank, with no evidence of blistering, targetoid lesions, or epidermal detachment (Figure 1).

Respiratory assessment showed tachypnea with oxygen saturations of 87–90% in ambient air, intercostal retractions, bilateral wheezing, and fine inspiratory crackles. Sinus bradycardia was also observed at presentation, with resting heart rates between 44 and 50 beats per minute. Pediatric cardiology evaluation confirmed the finding, with electrocardiography showing sinus rhythm at 48 bpm and normal conduction intervals and echocardiography demonstrating preserved biventricular function (left ventricular ejection fraction 72%) without structural abnormalities. On admission, the patient was hemodynamically stable, with warm extremities and normal blood pressure, and his heart rate increased appropriately to 86 bpm with activity such as mobilization or coughing, indicating an intact chronotropic response.

The laboratory results in this case of RIME (Table 1) show progressive leukocytosis with neutrophilia and persistent lymphopenia, consistent with an acute inflammatory response. Platelets increased steadily, suggesting reactive thrombocytosis, while hemoglobin declined slightly, reflecting possible inflammatory anemia. CRP decreased rapidly (23.8 → 1.5 mg/L), and D-dimers were only mildly elevated, supporting systemic inflammation. Overall, the findings indicate a transient, inflammation-driven disturbance with early resolution. Following transfer to our unit, a combined rapid antigen test for respiratory syncytial virus (RSV), influenza A and B, and SARS-CoV-2 was also performed; the result was positive for SARS-CoV-2 and negative for the other pathogens. For *Mycoplasma pneumoniae*, diagnosis was based on serological testing, which showed elevated IgM antibodies (>27.0 AU/mL; cut-off < 10.0 AU/mL) together with high IgG levels (>200 AU/mL; cut-off < 10.0 AU/mL). This antibody profile was compatible with a recent or ongoing infection. However, given the kinetics of antibody responses, the possibility of prior exposure with reinfection cannot be excluded. Screening for other potential infectious and autoimmune causes was performed. Tests included ASLO, anti-thyroid peroxidase antibodies, and viral serologies for Epstein–Barr virus, herpes simplex virus, and cytomegalovirus, all of which were negative.

**Figure 2:**
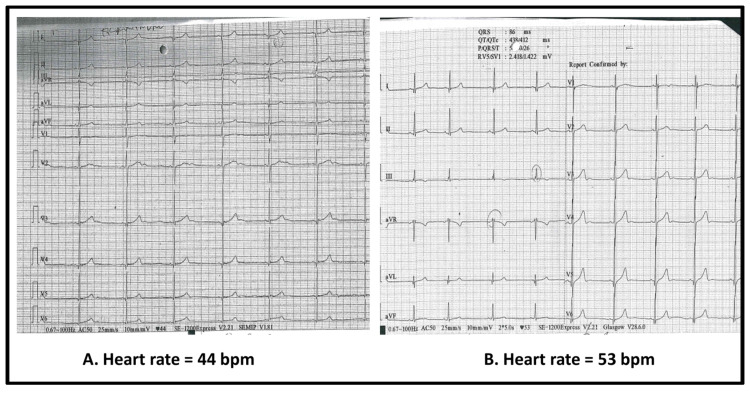
Twelve-lead ECG tracings from the same patient, showing sinus bradycardia with a ventricular rate of 44 bpm at baseline, day 1 (**A**), and subsequent improvement to 53 bpm on repeat recording, day 2 (**B**). Both demonstrate regular rhythm with narrow QRS complexes.

Serial ECGs (Figure 2) showed sinus bradycardia without conduction abnormalities. Chest radiography revealed bilateral interstitial infiltrates without focal consolidation. Echocardiography incidentally demonstrated a small right-sided pleural effusion, not detected radiographically, with preserved left ventricular function (EF 72%). Ophthalmologic evaluation confirmed mucopurulent conjunctivitis without corneal involvement. Bacteriology testing of conjunctival secretion was not performed, which we acknowledge as a limitation.

Overall, these findings supported a diagnosis of RIME, characterized by extensive mucosal involvement in more than two sites, minimal skin findings, confirmed *M. pneumoniae* infection, and exclusion of drug-induced or autoimmune causes. SARS-CoV-2 co-infection was considered an exacerbating factor.

Multidisciplinary care was provided. Empirical meropenem was initiated at admission (1 g intravenously three times daily for 5 days) after consultation with an infectious disease specialist, as approval is mandatory in our hospital for carbapenem therapy. Consultation with an ICU specialist was also obtained as part of the multidisciplinary approach. In parallel, azithromycin was started to cover a possible *Mycoplasma pneumoniae* infection, at a dose of 200 mg/5 mL, 10 mL orally once daily for 5 days. Because serological testing for *M. pneumoniae* had to be outsourced, results were delayed. Once infection was confirmed and the patient showed clinical improvement, meropenem was discontinued.

High-dose intravenous methylprednisolone (500 mg/day i.v. initially, followed by taper) was administered to mitigate severe mucosal and pulmonary inflammation. Given the acute respiratory failure, the patient was placed on low-flow oxygen via face mask whenever SpO_2_ dropped below 95%. Under this regimen, oxygen saturation stabilized around 97–98%, without the need for high-flow or advanced ventilatory support. Supportive care included i.v. fluids, oxygen supplementation, and inhaled bronchodilators. Gastroprotection was administered in parallel. Given the severe oral mucosal involvement and the risk of secondary fungal colonization, topical oral antifungal (nystatin/glycerin rinses) was given, and i.v. fluconazole was added early in the course. Netilmicin ophthalmic drops and eyelid hygiene were initiated due to bilateral conjunctivitis with mucopurulent discharge, consistent with bacterial superinfection.

Clinical improvement was observed over the course of hospitalization. Oral and ocular symptoms began to resolve by day 4, while respiratory function normalized progressively. Oxygen therapy and bronchodilator treatment were discontinued by day 6. Inflammatory markers returned to baseline. Bradycardia gradually resolved during hospitalization, coinciding with improvement in the underlying infection and normalization of systemic inflammatory markers; by discharge, the patient’s heart rate had returned to age-appropriate values, with no arrhythmias or residual cardiac dysfunction. The patient was discharged after 7 days in stable condition, with near-complete resolution of mucocutaneous lesions documented at follow-up 12 days post-discharge.

## 3. Discussion

### 3.1. RIME: Beyond Mycoplasma Pneumoniae

RIME is most often triggered by *Mycoplasma pneumoniae*, but an expanding body of literature has implicated other respiratory pathogens such as *Chlamydia pneumoniae*, influenza B, adenovirus, and, more recently, SARS-CoV-2 [8,9]. Several documented cases demonstrate that similar mucocutaneous syndromes—historically termed MIRM and now more broadly classified under RIME—can occur in the context of non**-***M. pneumoniae* or coinfections with other pathogens. The case series presented in Table 2 underscores the broad infectious triggers of RIME, with *Mycoplasma pneumoniae* as the most common, but several others—including influenza viruses, adenovirus, SARS-CoV-2, *Chlamydia pneumoniae*, and Group A *Streptococcus*—are also clearly implicated. The mucosal-predominant presentation remains a diagnostic hallmark, often involving oral, ocular, and genital sites, while cutaneous findings are variable and frequently minimal. Recognizing this pattern is essential to avoid misclassification as erythema multiforme or Stevens–Johnson syndrome. A particularly notable feature is the recurrent nature of RIME in some patients, often with different infectious triggers across episodes. This suggests a possible underlying predisposition and highlights the importance of longitudinal follow-up and anticipatory guidance for patients and families. Overall, these cases reinforce the value of thorough infectious workup in mucositis-predominant syndromes and the need to recognize RIME as a distinct clinical entity across age groups.

### 3.2. COVID-19 and the Evolving Immunopathogenesis of RIME

The COVID-19 pandemic has added diagnostic and therapeutic complexity to mucocutaneous syndromes like RIME, as SARS-CoV-2 may serve either as a primary trigger or as an immunologic amplifier in the context of co-infection [15,16].

Several pediatric teams across Europe have reported a marked resurgence of *Mycoplasma pneumoniae* infections in children following the COVID-19 pandemic, with incidence levels rising well above those observed in the pre-pandemic years [17]. This trend has been mirrored in Romania, where Ulmeanu and colleagues recently analyzed 63 pediatric cases of *M. pneumoniae* pneumonia hospitalized in 2024 [18]. Notably, their cohort also included two patients who developed reactive infectious mucocutaneous eruption (RIME), underscoring not only the significant increase in case numbers but also the expanding spectrum of clinical manifestations now being encountered. These observations support the growing recognition that the post-pandemic epidemiology of *M. pneumoniae* is characterized by both higher infection rates and more frequent extrapulmonary involvement. Our own experience is consistent with this pattern, reinforcing the need for heightened vigilance and early recognition of atypical presentations in order to guide timely management.

Though classically a respiratory virus, SARS-CoV-2 has demonstrated broad immunologic effects, including mucocutaneous manifestations and systemic inflammatory syndromes such as Multisystem Inflammatory Syndrome in Children (MIS-C) [19,20]. In this case, the concurrent presence of *M. pneumoniae* and SARS-CoV-2 likely contributed to a heightened immune response, resulting in more extensive mucosal inflammation. Both pathogens are capable of driving systemic immune activation through mechanisms such as cytokine release and immune dysregulation. Their co-occurrence may have produced a synergistic effect, consistent with the growing view that RIME represents a shared immunopathologic endpoint rather than a disease entity limited to *M. pneumoniae* alone [8,9]. These findings support the importance of maintaining a broad differential diagnosis in patients with mucositis, particularly in the setting of co-infection. Clinicians should be aware that viral pathogens, alone or in combination with bacterial agents, may alter disease severity, prolong symptom duration, and influence therapeutic response.

The pathophysiology of RIME has not been fully elucidated. Recent reports suggest a key role for polyclonal B cell activation, production of antibodies against *M. pneumoniae*, immune complex deposition, and keratinocyte apoptosis. Da Silva et al. [21] emphasized this mechanism as the basis for mucosal inflammation and epithelial damage in MIRM. Although complement levels were not measured in our patient, which represents a limitation, the clinical constellation aligns with an immune-mediated rather than direct infectious process.

Recent reports also describe cases of RIME following COVID-19 infection in vaccinated individuals, raising questions about the role of vaccine-modulated immunity in the development of post-viral mucocutaneous syndromes [22]. Although vaccination remains critical to mitigating severe disease, these cases suggest that it may not fully prevent immune-mediated sequelae. The immunological interplay between vaccine-primed responses and subsequent viral infection is not yet well understood and warrants further investigation.

### 3.3. Distinguishing RIME from Other Mucocutaneous Syndromes

RIME can be clinically challenging to distinguish from Stevens–Johnson syndrome (SJS) and toxic epidermal necrolysis (TEN), both of which share mucosal involvement and may present similarly at onset. However, key differences in etiology, clinical course, and prognosis are important to recognize. Unlike SJS/TEN—typically drug-induced and associated with epidermal necrosis—RIME is triggered by infection and often follows a more self-limited trajectory [7,23,24]. Careful history-taking and an awareness of preceding infections are essential for accurate diagnosis and appropriate management. Table 3 summarizes the key clinical and etiologic features that help differentiate RIME from other mucocutaneous syndromes with overlapping presentations [7,25,26,27,28].

### 3.4. Clinical Management of RIME—Challenges and Considerations

Therapeutic strategies for RIME remain heterogeneous. Antibiotic therapy targeting *M. pneumoniae* is widely accepted, with macrolides as first-line treatment. In contrast, the role of systemic corticosteroids remains debated. In our patient, high-dose methylprednisolone was initiated, given the extent of mucositis, ocular involvement, and respiratory compromise. This was combined with macrolide therapy, consistent with the principle that steroids may be beneficial in immune-mediated damage, provided adequate antimicrobial coverage is ensured. In this case, the patient’s significant improvement following initiation of high-dose corticosteroids alongside antimicrobial therapy highlights the potential value of early immunomodulation in severe RIME. Although systemic corticosteroid use in mucocutaneous disease has historically been approached with caution due to concerns about masking infections or provoking complications, emerging evidence and clinical experience suggest that they can be effective and safe when used judiciously [29,30,31]. Our patient experienced rapid resolution of mucositis and systemic symptoms without steroid-related adverse events, consistent with other case reports supporting early corticosteroid use in RIME with extensive mucosal involvement [29].

The decision to escalate to meropenem was made in consultation with an infectious disease specialist, whose approval is mandatory in our hospital for carbapenem use. Although laboratory findings on admission suggested a relatively mild profile, the patient’s clinical presentation was far more concerning, with acute respiratory compromise, systemic deterioration, and severe mucositis—circumstances that also prompted transfer to our facility. Clinical experience has taught us that the patient’s overall clinical condition must take precedence over reassuring laboratory values, which can sometimes be misleading. In this context, and following a multidisciplinary discussion between pediatrics and infectious disease specialists, empiric meropenem was initiated. An ICU specialist was also consulted as part of the multidisciplinary management. At the same time, azithromycin was added to cover a possible *Mycoplasma pneumoniae* infection, though confirmatory testing was delayed by 5 days due to the need for external processing. As a result, the patient received both antibiotics in parallel until meropenem was discontinued once serology confirmed *M. pneumoniae* and the patient had shown significant clinical improvement. In retrospect, earlier de-escalation—possibly continuing cephalosporin alongside targeted therapy—might have been more appropriate. This experience highlights the tension between severity-driven empiric decisions and stewardship obligations, reminding us that while decisions made in real time may appear justified, reflection afterward is essential for refining practice. Importantly, carbapenems are not recommended for routine management of pediatric community-acquired pneumonia in immunocompetent children outside ICU settings, as emphasized by international and national guidelines [32,33]. This case, therefore, underscores both the need to individualize therapy in severe presentations and the value of stewardship-based reassessment once the patient stabilizes.

A distinctive and educationally valuable feature of this case was the development of marked sinus bradycardia during the acute illness. While such bradycardia can be normal in well-conditioned adolescents, it was considered clinically significant in this case due to the patient’s acute systemic illness and associated respiratory compromise. Despite initial concern for myocarditis or conduction system disease, comprehensive cardiac evaluation revealed no structural abnormalities. Troponin levels were not measured, which represents a limitation; however, the patient remained hemodynamically stable, and bradycardia resolved spontaneously with clinical recovery, suggesting a transient, inflammation-related mechanism rather than myocardial injury. Although RIME-related mucocutaneous disease primarily affects the epithelium through inflammation, the broader range of extrapulmonary manifestations associated with *Mycoplasma pneumoniae* includes cardiac complications such as myocarditis and conduction abnormalities, including atrioventricular block [34,35]. While bradycardia has not been specifically reported in RIME, our patient’s presentation highlights the potential for cardiac involvement and suggests that monitoring may be warranted in severe or systemic cases. Although rarely reported, similar findings have been described in MIS-C and other pediatric COVID-19 presentations [36,37]. This reinforces the need for awareness of cardiac rhythm changes during systemic inflammation and supports conservative management in hemodynamically stable patients without cardiac dysfunction.

Taken together, this case highlights several important clinical lessons. We suggest the following considerations:

✓ RIME should be included in the differential diagnosis of mucositis, particularly following respiratory infections. Broad pathogen testing is essential when evaluating unexplained mucocutaneous eruptions.
✓ Co-infections, especially involving viral pathogens, can exacerbate immune-mediated mucocutaneous syndromes and should be actively evaluated. Clinicians should remain vigilant for viral reactivations, such as varicella-zoster virus, in the setting of systemic illness.
✓ Systemic corticosteroids may offer clinical benefit in severe cases, provided that appropriate antimicrobial coverage is ensured. However, their concomitant use with antimicrobial agents warrants careful monitoring, as immunosuppression may predispose to secondary infections or mask evolving complications. For this reason, the decision to initiate corticosteroid therapy should ideally involve multidisciplinary evaluation, and in some settings, additional authorization or oversight is required.
✓ Empirical escalation to carbapenems, including meropenem, should be reserved for selected severe pediatric cases and guided by infectious disease consultation. Timely diagnostic reassessment is critical, as delays can impact antimicrobial decision-making. Strict adherence to antimicrobial stewardship principles is essential to minimize unnecessary broad-spectrum use.
✓ Cardiac involvement, including bradycardia, should be monitored in the context of systemic illness. Unexplained bradycardia during systemic illness may be inflammatory in origin and, in the absence of structural heart disease, may not require intervention beyond monitoring.

## 4. Conclusions

This case highlights the complex nature of mucocutaneous syndromes like RIME triggered by co-infection with *Mycoplasma pneumoniae* and SARS-CoV-2. It provides valuable clinical insights into diagnosis, the role of immunomodulation, and transient sinus bradycardia. Though based on a single patient, these observations emphasize the multifaceted challenges of this syndrome. As respiratory infections evolve with emerging viruses like SARS-CoV-2, clinicians must broaden their awareness of immune-mediated complications. Further research is crucial to establish standardized diagnostic criteria and evidence-based management for RIME and related conditions. Focused efforts can help move from reactive management to proactive prevention and precision medicine in RIME by promoting early recognition through standardized diagnostic criteria, implementing pathogen-directed therapies, identifying predictive biomarkers for disease severity, and advancing individualized immunomodulatory approaches supported by multicenter registries.

## Figures and Tables

**Table 1:** Evolution of hematologic and biochemical parameters.

Parameter	Reference Range	Baseline (Regional Hospital)	Re-Evaluation (Regional Hospital)	Admission (Day 1, Our Center)	Follow-Up (Day 3)
**Leukocytes** (×10^3^/µL)	3.70–9.50	7.47	10.36	10.9	14.17
**Neutrophils** (×10^3^/µL)	1.80–8.00	5.09	6.41	7.9	11.87
**Lymphocytes** (×10^3^/µL)	2.00–6.50	1.63	2.62	1.87	1.52
**Monocytes** (×10^3^/µL)	0.20–1.00	0.72	1.14	1.0	0.75
**Erythrocytes** (×10^6^/µL)	4.30–6.00	4.71	4.97	4.5	3.68
**Platelets** (×10^3^/µL)	150–450	212	273	359	410
**Hemoglobin** (g/dL)	13.0–18.0	13.8	14.5	13.4	12.7
**Alanine aminotransferase (ALT)** (U/L)	0–55	14	17	16	20.6
**Aspartate aminotransferase (AST)** (U/L)	5–34	39	35	25	20.6
**Urea** (mg/dL)	15–36	22	27	29.96	30.9
**Creatinine** (mg/dL)	0.72–1.25	0.7	0.63	0.47	0.46
**C-reactive protein (CRP)** (mg/L)	0–5	23.83	12.26	5.5	1.52
**D-dimers** (mg/L FEU)	0–0.5	—	0.98	0.82	—

**Table 2:** Overview of recent postinfectious RIME cases in children.

Authors (Year)	Patient Profile	Pathogens	Mucosal Involvement	Skin Findings	Treatment Approach	Outcome
**Mayor Ibarguren et al. (2017)** [8]	6-year-old male	*Chlamydia pneumoniae*	Oral and genital erosions	No rash	Antibiotics + systemic corticosteroids	Full recovery
**Goyal & Hook (2019)** [10]	16-year-old male	*M. pneumoniae + Influenza B*	Oral, genital, ocular mucositis	Discrete erythematous papules on the extremities	Antibiotics + steroid eye drops	Full recovery
	16-year-old female	*M. pneumoniae + Influenza B*	Oral, genital, ocular mucositis	No rash	Antibiotics + topical and systemic corticosteroids + vaginal antifungals	Full recovery
**Gámez González et al. (2021)** [11]	7- and 14-year-old males	*Adenovirus*	Severe oral and ocular mucositis	Targetoid lesions; <10% BSA	IVIG + systemic corticosteroids + Ganciclovir	Full recovery
**Mazori et al. (2020)** [12]	8-year-old female	*GAS + M. pneumoniae* (1st episode)	Severe oral, ocular, and vulvar mucositis	Mild cutaneous involvement	Antibiotics + IVIG + systemic corticosteroids	Recurrent RIME (3 episodes) Full resolution after each episode
		*M. pneumoniae* (2nd episode)	Oral mucositis	No rash	Antibiotics + supportive care	
		*M. pneumoniae + Influenza B* (3rd episode)	Oral mucositis	No rash	Antibiotics + supportive care	
**Rodriguez et al. (2025)** [13]	5-year-old female	*Coronavirus NL63 + GAS*	Oral, conjunctival, nasal mucositis	Severe mucocutaneous involvement	Antibiotics + Acyclovir + IVIG + etanercept	Full recovery; resolution by day 10
**Falludi et al. (2025)** [14]	13-year-old male	*M. pneumoniae + Varicella-Zoster Virus*	Ocular, oral, and urogenital mucositis	Vesicular and targetoid lesions (limbs, trunk, earlobe); sparing scalp/palms/soles	Antibiotics + Acyclovir + IVIG	Full recovery, but resolution after several weeks
**Song et al. (2025)** [15]	13-year-old male	*M. pneumoniae* (1st episode)	Oral mucositis	Mild rash	Antibiotics	Recurrent RIME (4 episodes) Full resolution after each episode.
		*GAS* (2nd episode)	Oral mucositis	Mild rash	Antibiotics + topical corticosteroids	
		*Influenza A* (3rd episode)	Oral mucositis	Mild rash	Antibiotics + antiviral (Oseltamivir) + topical and systemic corticosteroids	
		*SARS-CoV-2* (4th episode)	Oral mucositis	Mild rash	Topical and systemic corticosteroids	
	18-year-old female	*M. pneumoniae*	Ocular, oral, and genital ulcerations	Vesicular lesions, maculopapular rash (face, trunk, arm)	IVIG + antibiotics + topical and systemic corticosteroids	Recurrent RIME (3 episodes) Full recovery; re-epithelialization over 3 months; required ocular amniotic membrane grafts
		*M. pneumoniae + Influenza A*	Oral and genital mucositis	Erythematous eroded papules (buccal mucosa, lips, clitoral hood)	IVIG + antibiotics + topical and systemic corticosteroids	Full recovery; resolution after 1 week
		Unknown infectious agent	Oral mucositis	No rash	IVIG + antibiotics + systemic corticosteroids	Full recovery; resolution after 2 weeks

**Abbreviations: BSA**—Body Surface Area; **GAS**—Group A *Streptococcus*; **IVIG**—Intravenous Immunoglobulin.

**Table 3:** Characteristic profiles of RIME, SJS/TEN, Erythema Multiforme, and Multisystem Inflammatory Syndrome in Children [7,25,26,27,28].

Feature	RIME	SJS/TEN	Erythema Multiforme (EM)	MIS-C
Etiology	Infectious triggers, primarily *Mycoplasma pneumoniae*; also, other respiratory pathogens (e.g., *Chlamydia pneumoniae*, influenza, adenovirus, SARS-CoV-2)	Drug-induced hypersensitivity reactions, commonly from sulfonamides, anticonvulsants, NSAIDs	Mostly HSV infection-associated; occasionally other infections	Post-infectious inflammatory syndrome after SARS-CoV-2 infection
Onset	Subacute; follows respiratory illness by days to weeks	Acute; usually 1–3 weeks after drug initiation	Acute; days after HSV infection	Delayed, 2–6 weeks after COVID-19 infection
Mucosal Involvement	Prominent and often severe; usually affects ≥2 mucosal sites (oral, ocular, genital)	Severe mucositis involving multiple sites (oral, ocular, genital), often painful	Mild to moderate mucositis, predominantly oral and sometimes ocular	Variable mucosal involvement; oral mucositis is common
Skin Findings	Minimal to mild rash; may include sparse targetoid or papular lesions	Dusky macules, confluent erythema, blistering, epidermal detachment	Typical “target” lesions, mostly acral (hands/feet), classic iris-shaped	Polymorphic rash: maculopapular, urticarial, or purpuric lesions
Systemic Involvement	Occasionally present	Common systemic symptoms: fever, malaise, risk of sepsis, multi-organ dysfunction	Usually mild or absent systemic symptoms	Prominent systemic inflammation with cardiac dysfunction, shock, GI symptoms
Epidermal Necrosis	Absent	Present	Absent	Absent
Course	Self-limited but may recur with new infections; favorable prognosis	Potentially life-threatening; requires hospitalization and supportive care	Self-limited, resolving in weeks; rare recurrence	Requires hospitalization, intensive care often needed; generally good outcomes with treatment
Prognosis	Excellent with supportive care; recurrence possible	Variable; SJS mortality ~10%, TEN up to 30%	Excellent; self-resolving in most cases	Generally good with timely treatment; potential for severe complications
Diagnostic Clues	Preceding respiratory infection; mucositis predominates; mild or absent skin rash	Recent drug exposure; epidermal detachment; severe mucositis with painful erosions	History of HSV; characteristic target lesions; mild mucositis	Recent SARS-CoV-2 infection; systemic inflammation; multiorgan involvement

**Abbreviations: SJS**: Stevens–Johnson Syndrome; **TEN**: Toxic Epidermal Necrolysis; **EM**: Erythema Multiforme; **MIS-C**: Multisystem Inflammatory Syndrome in Children; **HSV**: Herpes Simplex Virus; **GI**: Gastrointestinal, NSAIDs: Nonsteroidal Anti-Inflammatory Drugs.

## Data Availability

Data sharing is not applicable to this article.

## Abbreviations

The following abbreviations are used in this manuscript:

ASLO	Anti-streptolysin O
BSA	Body Surface Area
COVID-19	Coronavirus Disease 2019
DEN	Drug-induced Epidermal Necrolysis
ECG	Electrocardiogram
EF	Ejection Fraction
EM	Erythema Multiforme
ESR	Erythrocyte Sedimentation Rate
FiO_2_	Fraction of Inspired Oxygen
GAS	Group A *Streptococcus*
GI	Gastrointestinal
HSV	Herpes Simplex Virus
ICU	Intensive Care Unit
IgG	Immunoglobulin G
IgM	Immunoglobulin M
IV	Intravenous
IVIG	Intravenous Immunoglobulin
MIS-C	Multisystem Inflammatory Syndrome in Children
MIRM	Mycoplasma pneumoniae-Induced Rash and Mucositis
NSAIDs	Nonsteroidal Anti-Inflammatory Drugs
PCR	Polymerase Chain Reaction
RIME	Reactive Infectious Mucocutaneous Eruption
SARS-CoV-2	Severe Acute Respiratory Syndrome Coronavirus 2
SJS	Stevens–Johnson Syndrome
TEN	Toxic Epidermal Necrolysis
TORCH	Toxoplasmosis, Other agents, Rubella, Cytomegalovirus, Herpes simplex
